# Two types of colistin heteroresistance in *Acinetobacter baumannii* isolates

**DOI:** 10.1080/22221751.2020.1821584

**Published:** 2020-09-27

**Authors:** Yoon-Kyoung Hong, Hyunkeun Kim, Kwan Soo Ko

**Affiliations:** Department of Microbiology and Samsung Medical Center, Sungkyunkwan University School of Medicine, Suwon, Republic of Korea

**Keywords:** Colistin, heteroresistance, *Acinetobacter baumannii*, population analysis profiling, stability

## Abstract

In this study, we investigated the colistin heteroresistance patterns in *Acinetobacter baumannii* isolates. To identify colistin heteroresistance, population analysis profiling was performed for six *in vitro* colistin-susceptible *A. baumannii* isolates. Survival rates with and without prior exposure to colistin (at concentrations between 0 and 32 mg/L) were measured in media with and without colistin. Amino acid substitutions were also detected in colonies that survived in media with 4 mg/L colistin without further antibiotic treatment in six *A. baumannii* isolates. A stability test was also performed to investigate whether colistin resistance is maintained without antibiotic treatment. Although only three isolates showed typical colistin heteroresistance pattern, colistin-resistant populations were identified even without prior exposure to colistin in all *A. baumannii* isolates. Nearly all colonies of typical colistin-heteroresistant isolates (Type I heteroresistance) that survived after exposure to high colistin concentrations were found to be colistin-resistant, whereas no resistant colonies were identified in the other isolates (Type II heteroresistance). Stability tests showed that most of the surviving populations in media with 4 mg/L colistin without further antibiotic exposure failed to preserve resistance to colistin. Colistin-resistant populations also showed either no change in amino acid sequences, or diverse amino acid substitutions. We identified two types of colistin heteroresistance in *A. baumannii* isolates. Because Type I colistin-heteroresistant *A. baumannii* isolates could not be eradicated *in vitro* by high concentrations of colistin, differentiating two colistin heteroresistance types would be important for the treatment of *A. baumannii* infections using colistin.

## Introduction

*Acinetobacter baumannii* is a catalase-positive, gram-negative, and opportunistic pathogen responsible for life-threatening infectious disease conditions including pneumonia, sepsis, and meningitis [1]. Treating *A. baumannii* infections is challenging due to the remarkable ability of this pathogen to acquire antibiotic resistance [2]. *A. baumannii* has also been listed among Priority 1 pathogens for which new antibiotics are urgently needed by World Health Organization (WHO) in 2017 [3].

Increasing numbers of multidrug – or carbapenem-resistant *A. baumannii* infections have urged the use of polymyxin antibiotics (colistin and polymyxin B) for their treatment [4]. The bactericidal activity of colistin is based on disruption of the bacterial membrane and subsequent leakage of cellular content via electrostatic interactions between L-2,4-diaminobytyric acid (Dab) groups of colistin and the 1- and 4-phosphate groups of lipid A of the bacterial outer membrane [5,6]. *A. baumannii* may develop colistin resistance either through modification of lipid A with phosphoethanolamine associated with PmrAB two-component regulatory system or loss of lipooligosaccharide (LOS) [7–9].

Antibiotic heteroresistance is referred as a phenomenon where subpopulation of susceptible bacterial isolates exhibits reduced susceptibility to a particular antibiotic [10,11]. Heteroresistance to colistin in *A. baumannii* has been reported frequently [12–15]. These previous studies showed that colistin heteroresistance is common, and associated with PmrAB mutations, although the clinical significance has not been fully determined yet [12,15].

In this study, we reported two types of colistin heteroresistance in *A. baumannii* isolates. One is typical heteroresistance, a phenotype in which a bacterium includes subpopulations showing a substantial reduction in antibiotic susceptibility compared with the main population, has been identified in population analysis profiling (PAP), a gold standard method for determination of antibiotic heteroresistance [10]. In this type of heteroresistance, colistin-resistant colonies were identified in 10 mg/L of colistin concentration in PAP. In the other type of colistin heteroresistance, no surviving colonies were detected in high colistin concentration in PAP. However, colistin-resistant subpopulations could be identified even without prior exposure of colistin in these *A. baumannii* isolates. In addition, we investigated the effects of antibiotic treatment on the two types of colistin-heteroresistant *A. baumannii* isolates.

## Materials and methods

### Bacterial isolates

Six *A. baumannii* isolates, which were from blood of patients in Samsung Changwon Hospital in Korea (Table 1), were included in this study (Table 1). All of them were identified as colistin-susceptible via *in vitro* antibiotic susceptibility testing.

**Table 1.** Antibiotic susceptibility profiles and genotypes of *A. baumannii* isolates included in this study.

Isolate No.	Genotype	Minimum inhibitory concentration (MIC) (mg/L) ^a^											
		COL	A/S	CAZ	FEP	CIP	GEN	IMI	MER	SXT	*P*/T	T/C	TGC
Type I heteroresistance													
SCH2	ST681	1 (S)	2/1 (S)	16 (S)	8 (S)	0.25 (S)	1 (S)	0.25 (S)	0.5 (S)	2/38 (S)	**128/4 (R)**	16/2 (S)	0.5 (S)
SCH39	ST229	1 (S)	**32/16 (R)**	**64 (R)**	**64 (R)**	**4 (R)**	**16 (R)**	**16 (R)**	**16 (R)**	**32 /608 (R)**	**128/4 (R)**	**128/2 (R)**	1 (S)
SCH105	ST191	1 (S)	**32/16 (R)**	**64 (R)**	**64 (R)**	**4 (R)**	**16 (R)**	**16 (R)**	**16 (R)**	**16/1216 (R)**	**128/4 (R)**	**128/2 (R)**	2 (S)
Type II heteroresistance													
SCH91	ST191	1 (S)	**32/16 (R)**	**64 (R)**	**64 (R)**	**4 (R)**	**16 (R)**	**16 (R)**	**16 (R)**	**32/608 (R)**	**128/4 (R)**	**128/2 (R)**	2 (S)
Sch113	ST191	1 (S)	16/8 (I)	**64 (R)**	**64 (R)**	**4 (R)**	**16 (R)**	**16 (R)**	**16 (R)**	**16/304 (R)**	**128/4 (R)**	**128/2 (R)**	2 (S)
K20-B-871	ST191	2 (S)	**64/32 (R)**	**>64 (R)**	**>64 (R)**	**>64 (R)**	**>64 (R)**	**>64 (R)**	**>64 (R)**	**64/1216 (R)**	**>256/4 (R)**	**128/2 (R)**	2 (S)

^a^ CST, colistin; A/S, ampicillin/sulbactam; CAZ, ceftazidime; FEP, cefepime; CIP, ciprofloxacin; GEN, gentamicin; IMI, imipenem; MER, meropenem; SXT, trimethoprim/sulfamethoxazole; *P*/T, piperacillin/tazobactam; T/C, ticarcillin/clavulanate; TGC, tigecycline.

### *In vitro* antibiotic susceptibility testing

Antimicrobial susceptibilities of the six *A. baumannii* isolates were determined using the broth microdilution method as per the Clinical Laboratory and Standards Institute (CLSI) guidelines [16]. Susceptibilities to 12 antibiotics were tested, including colistin, ampicillin/sulbactam, ceftazidime, cefepime, ciprofloxacin, gentamicin, imipenem, meropenem, trimethoprim/sulfamethoxazole, piperacillin/tazobactam, ticarcillin/clavulanate, and tigecycline. Susceptibility was interpreted according to CLSI breakpoints [16], except for tigecycline. *Escherichia coli* ATCC 25922 and *P. aeruginosa* ATCC 27853 were used as quality control strains.

For each of the six *A. baumannii* isolates, ten colonies were selected, which survived in media with 4 mg/L colistin after no exposure to any antibiotics. For these strains, colistin minimum inhibitory concentrations (MICs) were determined using the same broth microdilution method as mentioned above.

### Population analysis profiling

To identify colistin heteroresistance in in the usual way, PAP was performed for the six colistin-susceptible *A. baumannii* isolates. Briefly, a 50 μL aliquot of bacterial culture containing 1.5 × 10^5^ cfu/mL was streaked onto Mueller-Hinton (MH) agar plates containing either 0, 0.5, 1, 2, 3, 4, 6, 8, or 10 mg/L colistin. Colonies were counted after overnight incubation at 37 °C. The limit of quantification (LOQ) was 20 cfu/mL. The PAP was repeated with three independent experiments.

### Measurement of survival rates after exposure to colistin

We performed the additional experiments to measure if a resistant subpopulation exists originally in the susceptible isolate, and if the resistant population survived against high concentrations of colistin. We expected that only typical heteroresistant isolates (designated Type I later) show existence of colistin-resistant subpopulation, but not others (designated Type II later) do not.

**Figure 1.**
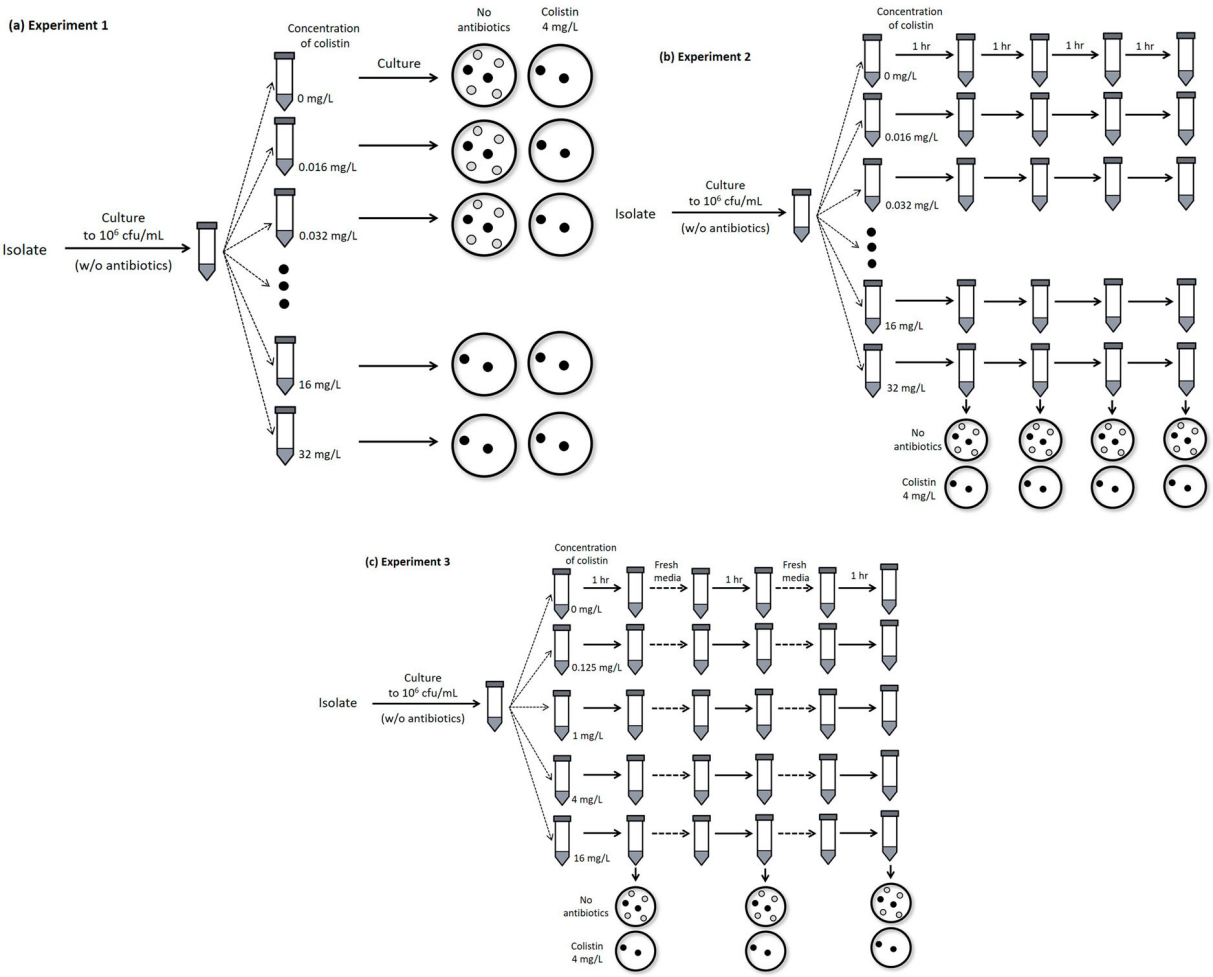
Illustrations of methods used to measure survival rates of total and colistin-resistant populations after exposure to various colistin concentrations. **(a, Experiment 1)** Bacteria were cultured in MH broth containing different colistin concentrations. After incubation for 18 h at 37°C with shaking, the total surviving colonies on MH agar plates without colistin and resistant colonies on plates with 4 mg/L colistin were counted. **(b, Experiment 2)** Unlike experiment 1, bacterial culture exposed to colistin was diluted every hour, and plated on MH agar with or without colistin. **(c, Experiment 3)** The bacterial culture was transferred to fresh media at each time point, and then cultivated.

*Experiment* 1. A total of 10 mL bacterial aliquot containing 1.5 × 10^5^ cfu/mL was inoculated into flasks including Mueller-Hinton (MH) broth and 0, 0.016, 0.032, 0.062, 0.125, 0.25, 0.5, 1, 2, 4, 8, 16, or 32 mg/L colistin (Figure 1a). The cultures were incubated for 18 h at 37°C with shaking. After incubation, the cultures were serially diluted using a saline solution, and cultured on agar plates containing MH broth with (4 mg/L, a breakpoint concentration of colistin resistance) or without colistin. Colonies were counted after 18 h of incubation at 37°C.

*Experiment* 2. Similar to experiment 1, 10 mL of bacterial aliquot was inoculated into flasks including MH broth and 0, 0.016, 0.032, 0.062, 0.125, 0.25, 0.5, 1, 2, 4, 8, 16, or 32 mg/L colistin. The cultures were incubated at 37°C, and diluted cultures were plated on MH agar with (4 mg/L) or without colistin every hour, unlike the procedure used in experiment 1 (Figure 1b). Colonies were counted after 18 h of incubation at 37°C.

*Experiment* 3. A similar procedure to that of experiment 2 was followed with several modifications. Here, the cultured bacteria were transferred to fresh media prior to plating (Figure 1c). The surviving colonies were counted after 1, 2, 3, 4, 8, and 12 h culture. Colistin concentrations in flasks were 0, 0.125, 1, 4, and 16 mg/L.

### TDtest for detection of antibiotic tolerance

The Tolerance Disk Test (TDtest) was performed to identify antibiotic tolerance in *A. baumannii* isolates [17]. First, Kirby-Bauer disk diffusion assay was performed using a disk on which colistin (10 mg/L) was applied. Next, this “colistin disk” was replaced with a “glucose disk” (2 mg/L glucose was applied) after 18 h, and the plate was incubated overnight. If newly growing colonies are observed within the inhibition zone, it is considered that tolerant subpopulations exist in the susceptible isolate.

### Determination of PmrCAB and LpxACD amino acid sequences

Ten colonies were obtained from cultures of each of the six *A. baumannii* isolates grown in media containing 4 mg/L colistin without prior exposure to any other antibiotics. In addition, three colonies were obtained from three Type I *A. baumannii* isolates grown in media containing 4 mg/L colistin after exposure to high colistin concentration (32 mg/L). For these colonies, nucleotide and corresponding amino acid sequences of *pmrCAB* and *lpxACD* genes were determined.

### Stability test for colistin resistance

We performed stability test for resistance to know if colistin-resistant subpopulations without genetic variations in well-known genes associated with colistin resistance preserve their colistin resistance phenotype. In six *A. baumannii* isolates, we selected each one colony with no amino acid substitutions in PmrAB and LpxACD sequences among ten colonies surviving in media with 4 mg/L colistin without prior exposure to colistin in the experiment 1. Colonies were repeatedly sub-cultured in the absence of colistin treatment to investigate the stability of the colistin resistance. Overnight cultures were diluted to 1:100 in fresh LB medium without colistin, and incubated with shaking (180 rpm) at 37°C for 24 h. We estimated colistin MICs for all serial sub-cultures.

### Experimental procedures for other bacterial species and antibiotics

For *Escherichia coli* and *Pseudomonas aeruginosa* strains, experiment 1 was performed. These strains were not heteroresistant to colistin (MIC 1 mg/L). For the *A. baumannii* strain SCH2, PAP and survival rate measurement after exposure to antibiotics (experiment 1) were performed using ciprofloxacin and imipenem.

## Results

**Figure 2.**
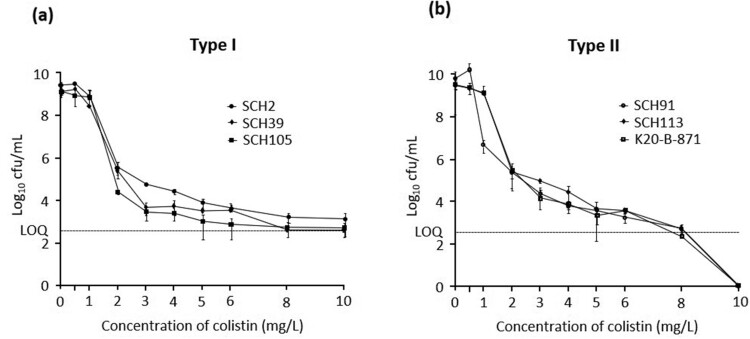
The results of population analysis profiling (PAP) of Type I colistin-heteroresistant *A. baumannii* isolates (SCH2, SCH39, and SCH105) (A) and Type II colistin-heteroresistant *A. baumannii* isolates (SCH91, SCH113, and K20-B-871) (B). LOQ: limit of quantification.

Six *A. baumannii* isolates were found susceptible to colistin based on broth microdilution assay results (MICs of 1 or 2 mg/L) (Table 1). However, population analysis profiling showed that cultures of three *A. baumannii* isolates showed typical heteroresistance pattern, in which subpopulations survived at a frequency of >10^−7^ in the media containing 10 mg/L colistin (Figure 2a). However, the other three isolates did not, that is, no colonies were found in the media with 10 mg/L colistin (Figure 2b). Typical colistin heteroresistance identified clearly in PAP was designated Type I. The others could not be identified in PAP, but it was shown that resistant subpopulation exists in original population without prior antibiotic exposure (described below). Thus, these should be regarded to be heteroresistant to colistin, and designated Type II. Sequence types of the typical colistin-heteroresistant isolates were different from each other (ST681, ST229, and ST191), whereas those of the other isolates were identical (ST191) (Table 1). Five *A. baumannii* isolates excluding an isolate SHC2 were resistant to most other antibiotics except for tigecycline (Table 1).

**Figure 3.**
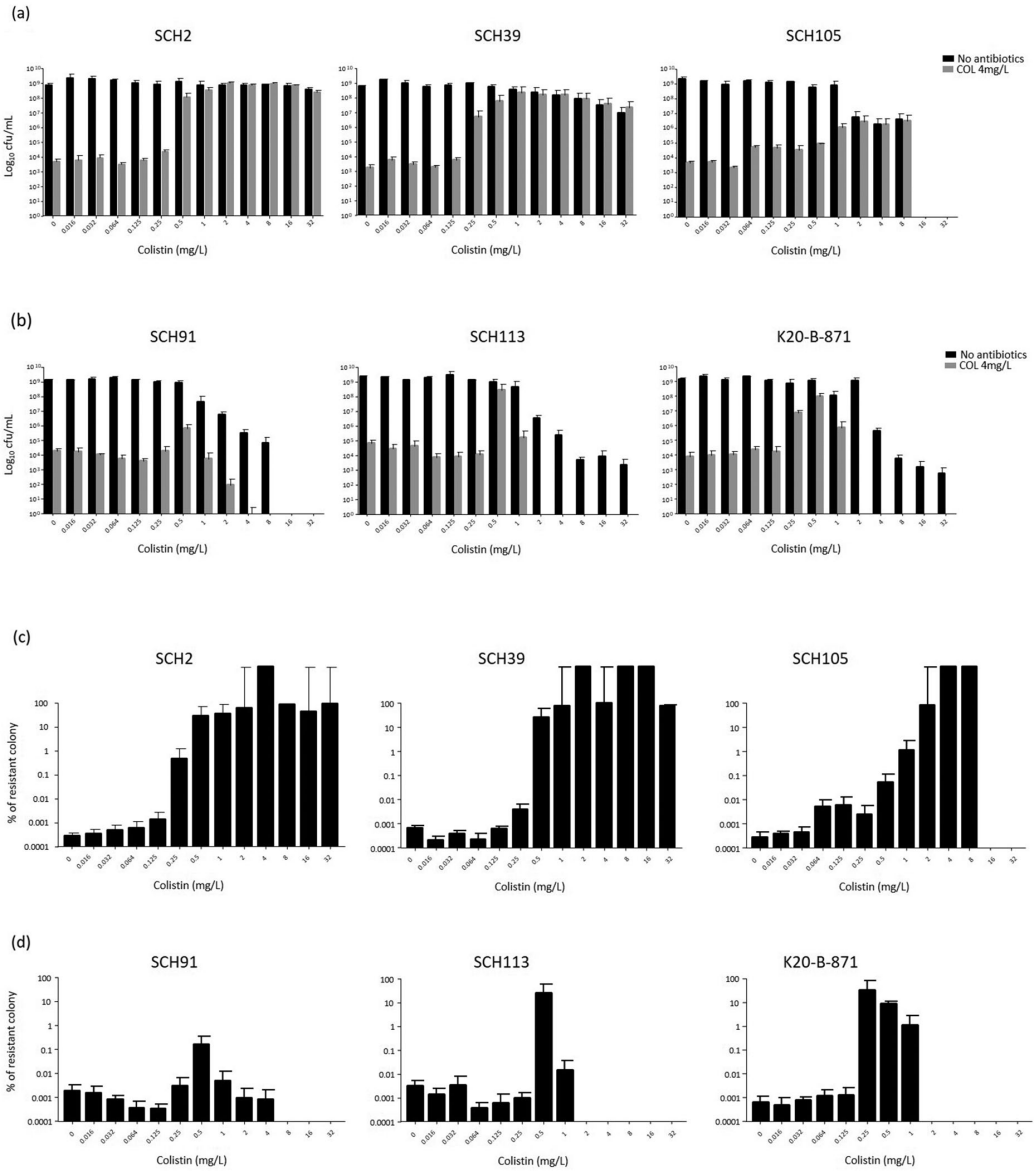
The results of experiment 1. Total and colistin-resistant populations with respect to colistin concentration. The total surviving populations were identified in media without antibiotics, and colistin-resistant populations were identified in media including 4 mg/L colistin. **(a)** The total and colistin-resistant, surviving populations in Type I *A. baumannii* isolates, **(b)** the total and colistin-resistant, surviving populations in Type II *A. baumannii* isolates, **(c)** the rates of colistin-resistant population within the total populations in Type I *A. baumannii* isolates, and **(d)** the rates of colistin-resistant population within the total populations in Type II *A. baumannii* isolates.

Figures 3(a and b) show the surviving numbers of total and colistin-resistant sub-populations in typical colistin-heteroresistant (Type I) and the other (Type II) isolates with respect to colistin concentrations, respectively, in the experiment 1. Figures 3(c and d) indicate the ratios of the colistin-resistant colonies over total colonies in Figures 3(a and b), respectively. Contrary to initial expectations, in all *A. baumannii* isolates, including those of the Type II isolates, colonies surviving in MH broth with 4 mg/L colistin were surprisingly identified in PAP analysis even without prior exposure to colistin. Hence, approximately 10^−7^–10^−6^ of the total population was found to be resistant to colistin (Figure 3c and d). The survival patterns of populations were similar to each other irrespective of pattern in PAP at low colistin concentrations (below 0.5 mg/L): only a small proportion of the surviving population was resistant to colistin. On the contrary, survival patterns differed between Type I and Type II isolates with increasing colistin concentrations. Type I isolates survived after exposure to high concentrations of colistin (at least 8 mg/L), and most surviving colonies were resistant to colistin (Figure 3a and c). However, total rate of surviving colonies decreased, and colistin-resistant colonies were not identified after exposure to high concentrations of colistin in cultures of Type II isolates (Figure 3b and d).

We also performed the procedure described in experiment 1 for colistin-susceptible *E. coli* and *P. aeruginosa* strains (Supplementary Figure S1). These strains showed similar results to those obtained with colistin-susceptible *A. baumannii* strains. Although these were not initially exposed to colistin, some colonies survived in media with 4 mg/L colistin. However, they did not survive upon exposure to high colistin concentrations. Experiment 1 was performed using ciprofloxacin and imipenem for SCH2 strain, which is susceptible to both antibiotics. PAP showed that this strain was not heteroresistant to any of these antibiotics (Supplementary Figure S2a). No ciprofloxacin – or imipenem-resistant colonies were identified at any concentration, including the case where no antibiotics were used. The total surviving population also decreased as antibiotic concentration increased (Figure S2b).

**Figure 4.**
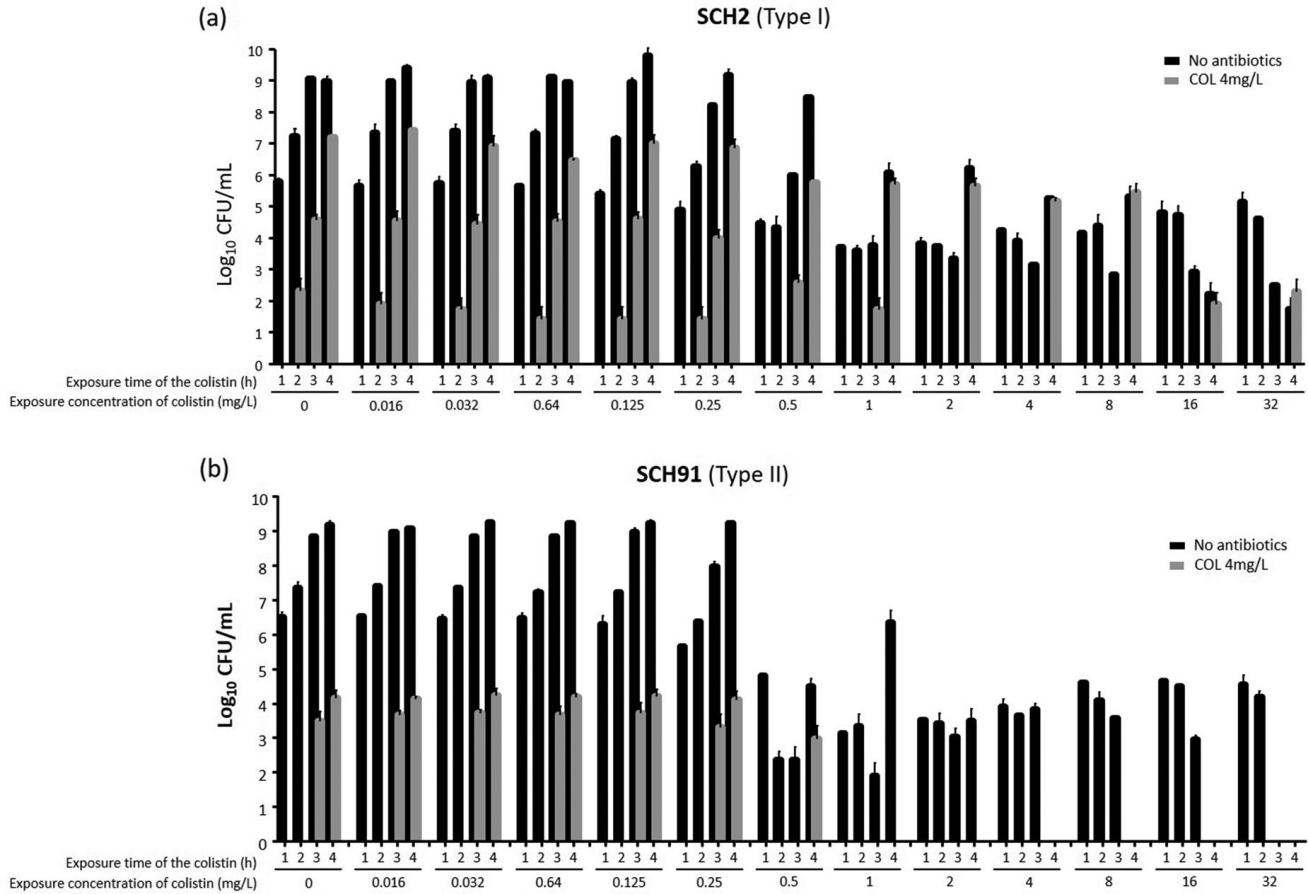
The results of experiment 2. Total and colistin-resistant populations with respect to colistin concentration. **(a)** SCH2, a Type I *A. baumannii* isolates, **(b)** SCH91, Type II *A. baumannii* isolates.

**Figure 5.**
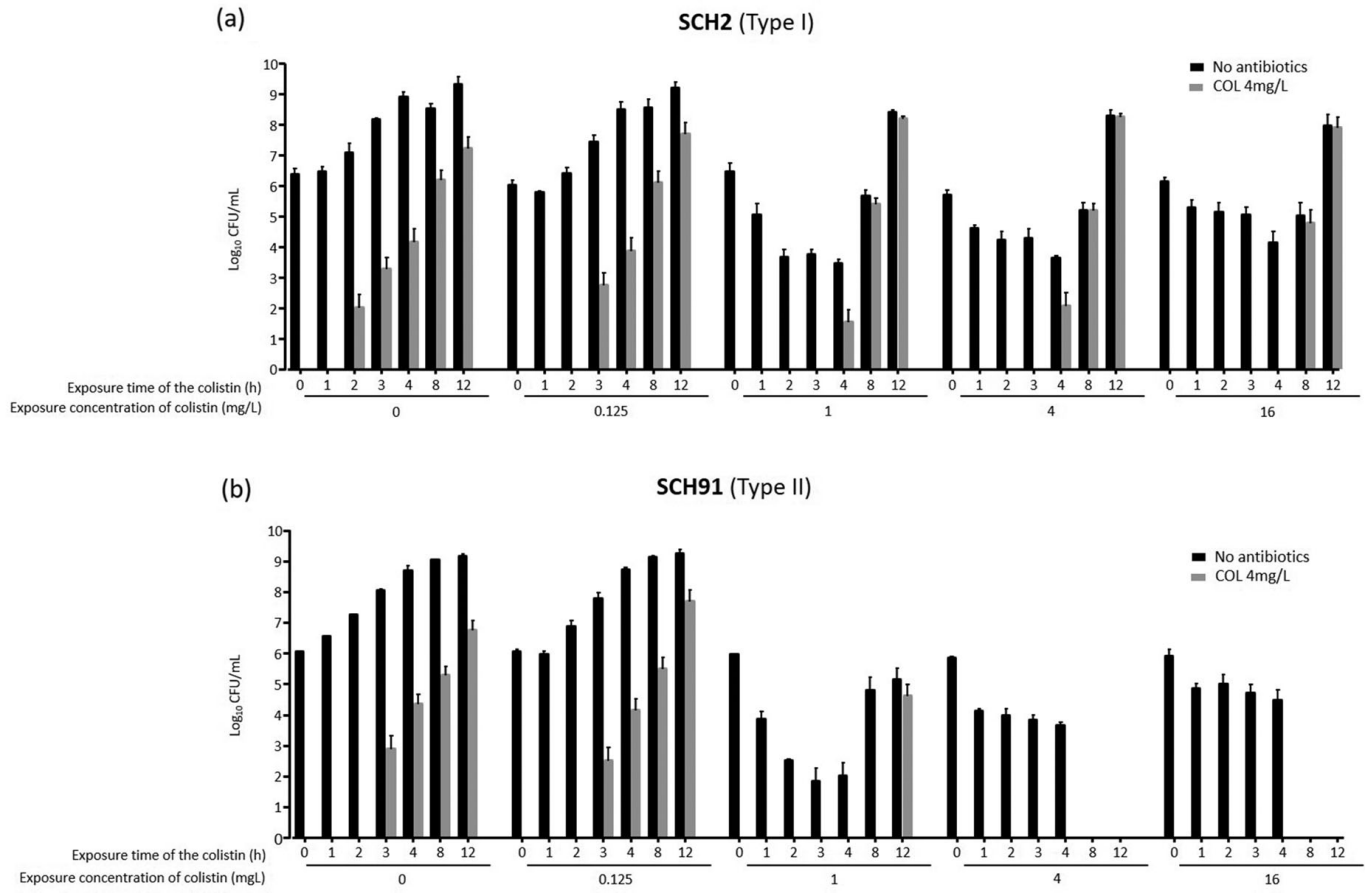
The results of experiment 3. Total and colistin-resistant populations with respect to colistin concentration. **(a)** SCH2, a Type I *A. baumannii* isolates, **(b)** SCH91, Type II *A. baumannii* isolates.

We performed additional experiments (experiments 2 and 3, as described in Methods) on survival rates after exposure to colistin to know if the survival rates change with exposure time of colistin in Type I and Type II colistin-heteroresistant isolates. Figures 4 and 5 show the results of experiments 2 and 3, respectively, revealing different patterns of survival rates in Type I and Type II colistin-heteroresistant isolates with exposure time of colistin. These figures display the survival patterns of total (no antibiotics in media) and colistin-resistant subpopulations with respect to colistin concentration and exposure time in cultures of Type I (SCH2) and Type II (SCH91) isolates, respectively. In experiment 2, the ratio of surviving colonies on 4 mg/L of colistin to the total CFU obtained without antibiotics in the SCH2 isolate (Type I isolate) increased with increased exposure time until the colistin concentration of 0.5 mg/L. After that, the ratio decreased with time as colistin concentration was increased (Figure 4a). No colistin-resistant subpopulation was observed with short exposure times: colistin-resistant colonies were identified only after 4 h at concentrations above 2 mg/L. In cultures of the SCH91, a type II isolate, colistin-resistant subpopulations appeared at later time points than those of the type I isolate. Total surviving colonies decreased with increasing exposure times at concentrations above 4 mg/L (Figure 4b).

The procedure followed in experiment 3 was different from that of experiment 2, in that the cultures were re-inoculated into fresh media every hour. The results of experiment 3 (Figure 5) were similar to those of experiments 1 and 2 (Figures 3 and 4). Here, the number of colistin-resistant colonies gradually increased with time following exposure of Type I and Type II isolates to colistin (Figure 5a and b). The proportion of colistin-resistant subpopulation within the total population increased in cultures of the Type I isolate (SCH2) with increasing exposure time and colistin concentrations (Figure 5a). Colistin-resistant colonies were identified in cultures of the Type II isolate (SCH91) at low colistin concentrations, yet absent when the isolate was exposed to 4 and 16 mg/L colistin (Figure 5b).

**Figure 6.**
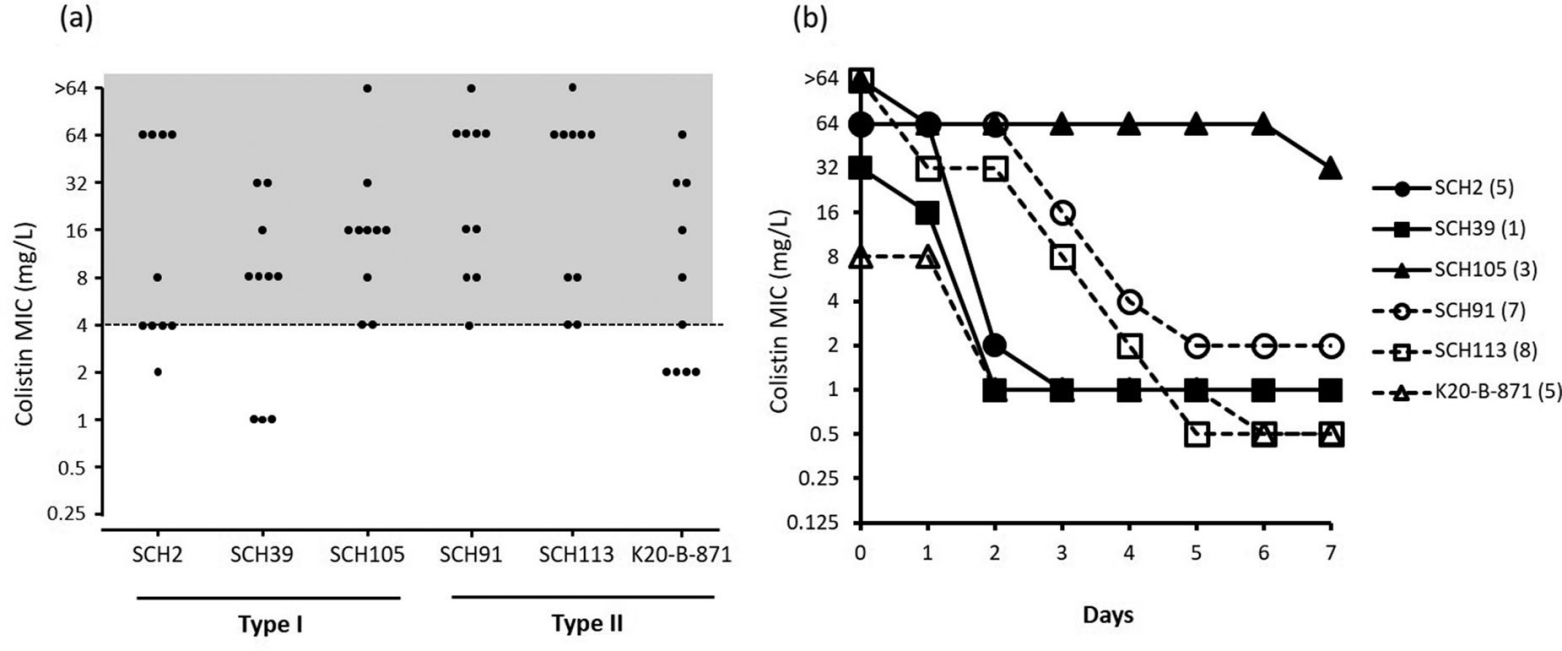
**(a)** Colistin MIC distributions of strains in media containing 4 mg/L colistin under exposure to colistin as described in experiment 1. The grey highlighted area indicates the MIC range corresponding to colistin resistance. **(b)** The results of stability test for colistin resistance.

MIC distribution of colonies that survived in media with 4 mg/L colistin without prior exposure to antibiotics in experiment 1 is shown in Figure 6(a). Most colonies of Type I as well as Type II isolates showed MICs equal to or higher than 4 mg/L, indicating colistin resistance. TDtest for detection of tolerant subpopulation yielded no growing colonies upon replacement of the colistin disk with a glucose disk (data not shown). The stability test showed that the colistin resistances in surviving colonies in media with 4 mg/L colistin without other antibiotic exposure were mostly unstable (Figure 6b). Here, only one colony of the Type I SCH105 isolate remained colistin-resistant even after seven days of serial sub-culturing in the absence of antibiotics.

Amino acid substitutions in PmrAB and LpxACD sequences were determined in colonies that survived in media with 4 mg/L without antibiotic exposure (Table 2). For some colonies, no amino acid substitutions were observed, whereas for others, observed substitutions were diverse. No amino acid alterations were observed in ten colonies of the Type I SCH2 isolate. Amino acid substitutions were identified in two and four colonies of isolates SCH39 and SCH105, respectively. Moreover, amino acid changes in PmrAB and LpxACD were observed in colonies of SCH39 and SCH105, respectively. There were no colonies exhibiting identical substitutions. All but two colonies of the Type II SCH91 isolate showed amino acid substitutions (six and two substitutions in PmrB and two in LpxC, respectively). Substitutions observed in these colonies were also different from each other. Three colonies of SCH113 and two of K20-B-871 isolates showed amino acid changes in PmrA and PmrB genes, respectively. Only three colonies of SCH113 isolate showed the same amino acid substitutions (S16L in PmrA).

**Table 2.** Amino acid substitutions in PmrCAB and LpxACD genes in colonies that survived in media with 4 mg/L colistin without antibiotic exposure.

Strain	Colony No.	Colistin MIC (mg/L)	PmrA	PmrB	LpxA	LpxC	LpxD
Type I heteroresistance							
SCH2	1	2					
	2	4					
	3	64					
	4	64					
	5	64					
	6	8					
	7	4					
	8	64					
	9	4					
	10	4					
SCH39	1	32					
	2	1					
	3	1					
	4	16					
	5	8					
	6	8					
	7	1					
	8	8	A14V				
	9	32		E301D			
	10	8					
SCH105	1	16			M169T		
	2	16					
	3	>64					
	4	32					
	5	8			R258H		
	6	4			T81M		
	7	16					T290I
	8	16					
	9	32					G231V
	10	17					
Type II heteroresistance							
SCH91	1	16					
	2	>64		T235I			
	3	16		L271F			
	4	64		P170L			
	5	4				M62I	
	6	8		L271F			
	7	64					
	8	64		S17R			
	9	8				C63Y	
	10	64		G260D			
SCH113	1	8					G309V
	2	8					
	3	32					
	4	32	S16L				
	5	4					
	6	4					
	7	32					
	8	>64					
	9	32	S16L				
	10	32	S16L				
K20-B-871	1	2					
	2	4					
	3	64		T232I			
	4	2					
	5	8					
	6	2					
	7	16					
	8	32					
	9	32		L168S			
	10	2					

Next, three colonies from each of the three Type I *A. baumannii* isolates were selected in media with 4 mg/L colistin after the exposure to high colistin concentration (32 mg/L). All of these strains were found to be resistant to colistin. Amino acid sequences of PmrAB and LpxACD genes were identical to each other in colonies of the same strain. No amino acid substitutions were observed in colonies of SCH2 isolate. On the contrary, substitutions were identified in colonies of other isolates: R263H in PmrB of SCH39 isolate, and A58V in LpxD of SCH105 isolate.

## Discussion

The occurrence of colistin heteroresistance in *A. baumannii* has been attributed to the development of antibiotic resistance and failures in therapeutic treatment [14,18]. The most important finding of this study was that there are two types of colistin heteroresistance in *A. baumannii* isolates. Antibiotic heteroresistance has been defined in various ways, with respect to clonality, level of resistance, frequency, and stability [10,11]. Mixed infections or slow-growing resistant mutants during antibiotic treatment may result in polyclonal heteroresistance. Level of resistance of resistant subpopulation compared with that of the main population may be different according to investigators. The frequency of the resistant population may also be different according to the definitions. Some investigators have defined heteroresistance as stable, but others have regarded it as heteroresistance even if the resistance of the subpopulation decreases or reverts to susceptibility [10]. No matter how heteroresistance is defined, it is obvious that heteroresistant isolates included a small resistant population in main susceptible population. Two types of colistin heteroresistance identified in this study may show different features. In the Type I heteroresistance, resistant populations are positively selected in the presence of a high concentration of colistin and expand during growth. On the other hand, resistance populations in the Type II heteroresistance show their resistance phenotype only at low antibiotic concentrations possibly due to mutations that confer some degree of antibiotic resistance but at the same time, they also impose a trade-off. In the absence of selection, these mutants are rapidly wiped out of the population. This Type II heteroresistance may be antibiotic-specific.

In this study, all *A. baumannii* isolates, which were judged as susceptible to colistin by method of determining MICs, are shown to include colistin-resistant populations without prior exposure to colistin. However, one group (Type I) showed a typical pattern of heteroresistance, but another group (Type II) did not. In addition, the pattern of surviving colonies with increasing concentration of exposure was different between two types. The proportion of colistin-resistant population was approximately 10^−5^ in all isolates, and this proportion was maintained until the isolates were exposed to colistin at 0.25–0.5 mg/L concentrations. While nearly only colistin-resistant population survived even with prior exposure of high concentration of colistin in Type I heteroresistant isolates, no resistant population survived with prior exposure of high concentration of colistin in Type II isolates.

The presence of Type II heteroresistance, which is defined as no survival of resistant population with exposure with high concentration of antibiotics, was demonstrated for other species as well (*E. coli* and *P. aeruginosa*). However, this phenomenon was not observed with other antibiotics such as fluoroquinolones and carbapenems, and therefore, may be a unique feature of colistin. Thus, treatment of even low concentrations of colistin increased the likelihood of the selection of resistant subpopulations regardless of bacterial species. The results presented in this study are also in line with those of a previous study on mutant prevention concentrations (MPCs) of colistin for *A. baumannii* and other gram-negative pathogens, where very high concentrations were reported [19].

On the contrary, treatments with high colistin concentration resulted in different results for Type I and II *A. baumannii* isolates. In Type I heteroresistant isolates, nearly the whole surviving population was found to be resistant upon exposure to high colistin concentrations, suggesting that colistin treatment on the Type I colistin-heteroresistant isolates may trigger the emergence of colistin resistance. However, no colistin-resistant colonies could be identified for Type II isolates at high colistin concentrations.

Our findings suggest that colistin may be ineffective for treatment of infections caused by Type I colistin-heteroresistant *A. baumannii* isolates. However, high doses of colistin may be effective against Type II colistin-heteroresistant *A. baumannii* infections. Thus, differentiating Type I heteroresistance from Type II heteroresistance is important for the treatment of gram-negative pathogen infections using colistin.

On the contrary, a high nephrotoxicity risk has also been previously reported for colistin treatment [20,21]. Intracellular accumulation of colistin was proposed to be a precondition for colistin-mediated renal damage, and high colistin doses increase the risk of nephrotoxicity [22]. Further considerations may be required to overcome these problems, for example usage of polymyxin B instead of colistin, antibiotic combinations, or additional treatments using antioxidant substances [23,24].

The colonies that survived in colistin-containing media showed different amino acid substitutions, even from the same isolate. Only two amino acid alterations (P170L and S17R in PmrB) have been reported in previous studies [25], but others including mutations in LpxACD were newly identified in this study. Although it is required to investigate if the mutations would be associated with colistin resistance, diverse colistin-resistant subpopulations of the original isolates may have been present prior to the start of experiments. In terms of stability of resistance, colistin-resistant subpopulations within the original isolates were different from those that acquired resistance following exposure to colistin [26]. The stability of the resistance in diverse subpopulations of the original isolates were likely unstable [10], and therefore, most of these subpopulations disappeared as antibiotic concentrations were increased. In contrast, the stabilities of resistance of some colistin-resistant subpopulations in Type I colistin-heteroresistant isolates were high.

In this study, we identified two types of colistin heteroresistance in *A. baumannii* isolates, in which colistin-resistant subpopulations were identified even without prior exposure to colistin. In Type I colistin-heteroresistant *A. baumannii* isolates, resistant subpopulations were selected and nearly all surviving bacteria were found to be colistin-resistant upon exposure to high colistin concentrations. In contrast, resistant subpopulations in Type II colistin-heteroresistant *A. baumannii* isolates disappeared at high concentrations of colistin. Furthermore, the resistance of subpopulations in both types may also be unstable. These results suggest that different treatment strategies against *A. baumannii* infections may be required based on the type of colistin heteroresistance.

## Supplementary Material

Supplementary_figure_2.JPG

Supp_Figure_final.jpg
