# Supplementary figures and images for: Two types of colistin heteroresistance in Acinetobacter baumannii isolates
Source: Emerg Microbes Infect. 2020 Sep 27;9(1):2114–23. doi: 10.1080/22221751.2020.1821584 (PMC7534296; doi:10.1080/22221751.2020.1821584)

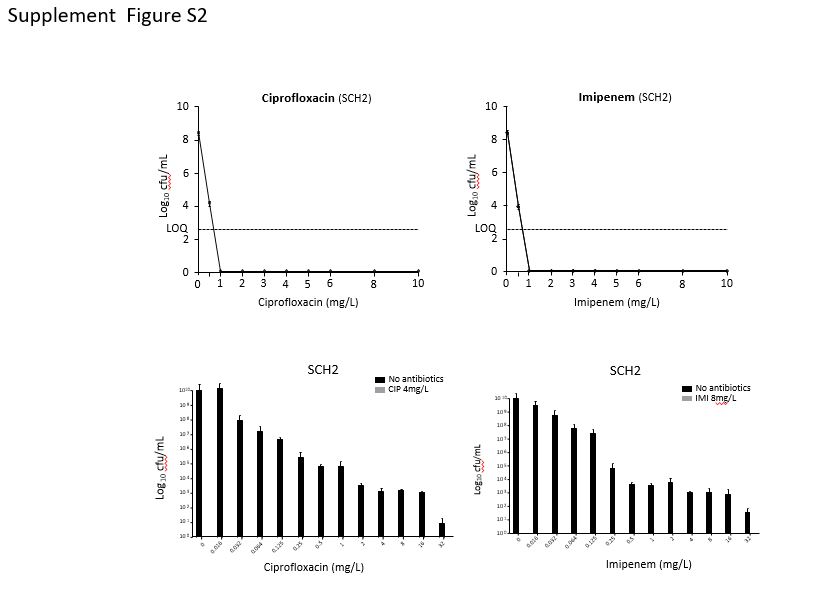

Supplement: Supplementary_figure_2.JPG [file TEMI_A_1821584_SM9146.jpg]

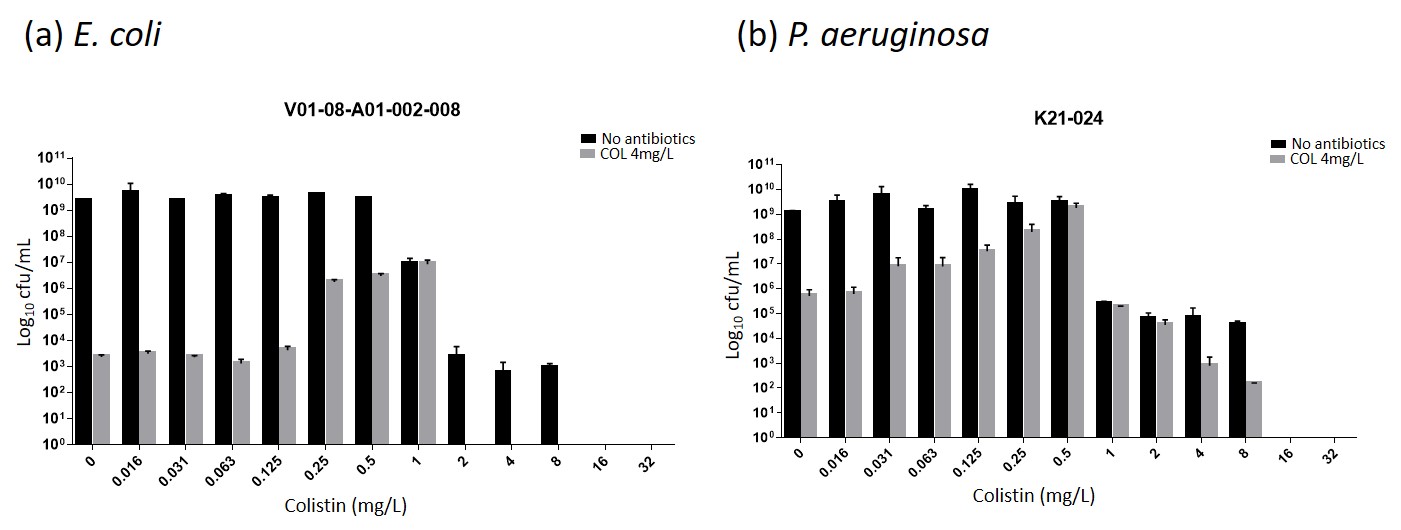

Supplement: Supp_Figure_final.jpg [file TEMI_A_1821584_SM9145.jpg]
